# Case report: Case series of isolated acute pericarditis after SARS-CoV-2 vaccinations

**DOI:** 10.3389/fcvm.2022.990108

**Published:** 2022-08-17

**Authors:** Marco M. Ochs, Markus Haass, Saskia Hagstotz, Sorin Giusca, Grigorios Korosoglou

**Affiliations:** ^1^Theresienkrankenhaus, Department of Cardiology, Angiology, and Intensive Care, Mannheim, Germany; ^2^Department of Cardiology, GRN Hospital Weinheim, Weinheim, Germany; ^3^Weinheim Imaging Center, Hector Foundation, Weinheim, Germany; ^4^University of Heidelberg, Heidelberg, Germany

**Keywords:** isolated pericarditis after SARS-CoV-2 vaccination, SARS-CoV-2 vaccination, acute isolated pericarditis, cardiac troponins, late gadolinium enhancement, T1 and T2 mapping

## Abstract

During the worldwide ongoing immunization campaign against SARS-CoV-2, growing data on very rare but potentially harmful side effects of such vaccines arise since approval trials have not been adequately powered to detect those events. Besides the already reported vaccine-related myocarditis, which primarily occurs in young male individuals, our attention was recently drawn to a series of older male and female patients, who were referred to our institutions with isolated acute pericarditis without myocardial damage, shortly after SARS-CoV-2 vaccination. We describe a series of five adult patients presenting with chest pain, shortness of breath and isolated pericarditis with and without pericardial effusion after SARS-CoV-2 vaccination. All patients underwent echocardiography and cardiac magnetic resonance, and the corresponding findings, including late gadolinium enhancement (LGE) and T1 and T2 mapping are reported herein. To our knowledge, such cases have not been systematically reported in the current literature so far.

## Introduction

More than 11.4 billion doses of SARS-CoV-2 vaccines have been administered worldwide by March 2022 during the largest immunization campaign in human history (1). Studies have undoubtedly proven the benefits of European Union authorized SARS-CoV-2 vaccines in terms of mortality and morbidity, thus outweighing the potential risks of this clearly life-saving strategy (2–4). However, clinical trials were typically underpowered to detect very rare adverse events after SARS-CoV-2 vaccination. Therefore, the continuous evaluation of potential side effects after SARS-CoV-2 vaccination, addressing risk-benefit evaluations, which may in the future guide our vaccination strategy is of major medical and scientific interest. While vaccine-related myocarditis was recently identified as a very rare adverse event in predominantly young men (5, 6), reports on the prevalence and characteristics of acute isolated pericarditis after SARS-CoV-2 vaccination have been limited so far.

In this dual-center study, we systematically report the demographic and clinical characteristics of five consecutive patients, who presented with cardiac symptoms related to possible perimyocarditis after SARS-CoV-2 vaccination and were diagnosed with isolated pericarditis after review of clinical data, laboratory markers, ECG changes, echocardiography, and cardiac magnetic resonance (CMR) findings, including late gadolinium enhancement (LGE) and T1 and T2 mapping. In all cases cardiac troponins were within normal range, and myocardial involvement was not detected by LGE or by mapping techniques.

## Materials and methods

In this dual-center report of two German cardiac imaging centers at the GRN Hospital, Weinheim and the Theresien Hospital, Mannheim we systematically reviewed our clinical data management systems, including patients who presented with cardiac symptoms, suggestive of acute perimyocarditis after SARS-CoV-2 immunization, using European Union authorized vaccines. All available clinical data on timing and type of the administered SARS-CoV-2 vaccine, potential prior SARS-CoV-2 infections, previous medical history, laboratory results and demographics were extracted from medical records and analyzed. Study participants individually consented for the anonymized data analysis as approved by our local ethics board (S-526-2016), and in accordance with the declaration of Helsinki.

All examinations were performed using 1.5 T MR systems (Siemens MAGNETOM Aera, Siemens Healthcare Erlangen, Germany). A standard protocol was used, according to previous recommendations (7). In short, an overview the of thorax is acquired (T2-HASTE–half-Fourier acquisition single shot turbo-spin echo), followed by three scout images. The cine acquisitions (steady state free precession) were performed in a stack of short axis covering the entire length of left ventricle (LV) and three long axes. T1 and T2 mapping were performed using standard short-axis mid-ventricular acquisition–MOLLI 5(3)3 and trueFISP sequences, respectively. The late gadolinium enhancement (LGE) acquisitions were performed in three long axis and multiple short axis covering the entire length of the left ventricle after the administration of (Dotarem^®^- gadoterate meglumine in a dosage of 0.1 mmol/kg) (7). Consensus interpretation was performed by at least two experienced cardiologists, specialized and board certified for CMR.

## Case series

A total of 44 patients were referred to our departments between December 2021 and February 2022 for CMR due to clinical suspicion of myocardial injury after SARS-CoV-2 vaccination, which comprises 12.5% of all CMR examinations performed in both centers during this time.

**Figure 1:**
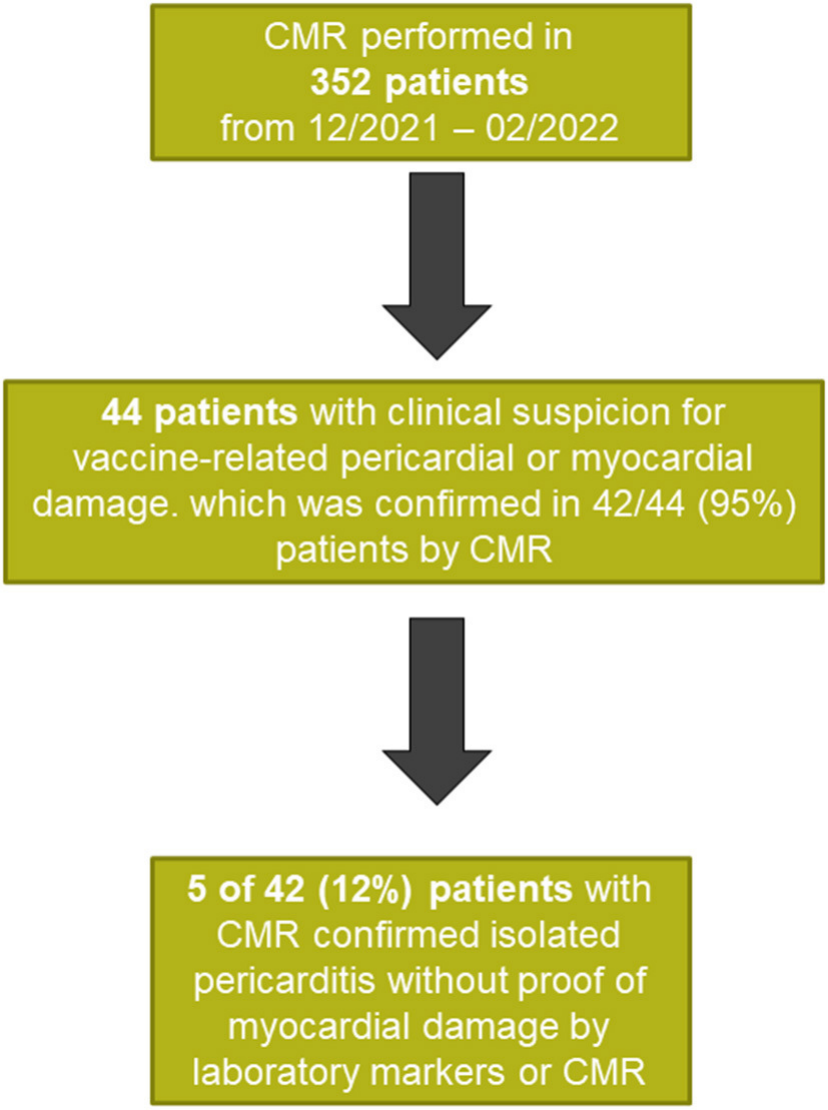
Study flow chart.

Five patients with a mean age of 55years (range 43–76 years) were diagnosed with vaccine associated isolated pericarditis, without proof of myocardial damage. A corresponding flow chart is presented in Figure 1.

Patients reported increasing chest pain and dyspnea or fatigue, whereas one patient also suffered from palpitations. In all cases, symptoms were associated with the second or third dose of mRNA SARS-CoV-2 vaccines with a time range of 2.8–7.0 days (median of 3.0 days) between the vaccination and onset of symptoms. Booster vaccination was performed using m-RNA vaccines with BioNTech in 4/5 patients (80%) or BioNTech and Moderna in one patient (20%) (Table 1).

**Table 1:** Patient characteristics.

**1**	**Case #1**	**Case #2**	**Case #3**	**Case #4**	**Case #5**	**Median and ranges**
Age (years)	75	55	78	42	43	55 (43–76)
Gender	Male	Female	Female	Male	Female	2/5 male
Risk factors	None	None	None	Type 2 DM	None	
BMI (kg/m^2^)	31.2	23.1	24.9	31.8	20.1	24.9 (22.4–31.4)
Type of vaccine	1. Astra Zeneca 2. BioNTech 3. Moderna	3*BioNTech	3*BioNTech	3*BioNTech	3*BioNTech	100% including m-RNA vaccines
Cardiac symptoms	Chest pain and dyspnea	Chest pain and dyspnea	Chest pain and dyspnea	Chest pain and dyspnea	Fatigue and dyspnea	(4/5) 80% chest pain and dyspnea
Days between last vaccination and symptoms	2	3	3	7	7	3.0 (2.8–7.0)
Days between symptoms onset and presentation	6	12	14	58	35	14 (11–41)
Highly sensitive troponin T (ng/L)	15.1	9.9	12.1	8.4	3.0	9.9 (7.1–12.9)
C-reactive protein (mg/L) (normal range <5)	73.9	224.8	237.4	4.8	0.5	73.9 (3.7–227)
White blood cell count (1,000/μL)	8.3	24.0	11.0	7.4	8.4	8.4 (8.0–14.2)
Pleural effusion (bilateral)	yes	yes	yes	no	no	(3/5) 60%
Pericardial effusion	yes	yes	yes	no	no	(3/5) 60%
ECG changes	Negative T-waves in V3-V6	Negative T-waves in I. II and aVF	None	Negative T-waves in V3–6	Negative T-waves in II. III& aVF	4/5 (80%) with significant ECG changes
LVEF (%)	52	71	68	61	65	65.0 (58.8–68.8)
T1/T2 values (ms) Normal ranges T1/T2 (900–1,080 ms/44–62 ms)	1,000/42	1,050/49	1,030/53	990/46	995/47	1,000 (993–1,035) for T1 47 (45–50) T2
Pericardial LGE	Diffuse/circular	Diffuse/circular	Diffuse/circular	Anterior and apical	Anterior and lateral	Diffuse in 3/5 cases Regional in 2/5 cases
Myocardial LGE	None	None	None	None	None	0/5
Treatment	Colchicine (3 months) and ibuprofen (2 weeks)	Colchicine (3 months). pericardial paracentesis. Cortisone	Colchicine (3 months). Ibuprofen (2 weeks). Cortisone	Colchicine (3 months)	Colchicine (3 months)	Colchicine in all. Paracentesis in 1/5. Cortisone in 2/5

*Five adults (age 58 ± 15 years) presented with clinical symptoms of acute pericarditis, all exhibiting pericardial late gadolinium enhancement (LGE) by CMR. The complaints began with all patients in close temporal association to the third dose of SARS-CoV-2 vaccines. None of the patients had evidence of acute myocardial damage by troponins or LGE. Four of five patients were treated with immunosuppressive therapy. In a single case urgent pericardiocentesis was necessary due to pericardial tamponade*.

Four of 5 patients had no atherogenic risk factors, whereas one had history of type 2 diabetes mellitus (Table 1). Patients #1–3 presented with acute symptoms within 2 weeks, whereas patients #4 and #5 presented 1–2 months after onset of cardiac symptoms. None of the patients have been pre-treated with non-steroidal anti-inflammatory drugs (NSAID), corticosteroids or colchicine before their initial presentation in our departments. The time range between symptom onset and presentation in our departments was 11–41 days (median of 14 days). ECG showed abnormalities in 80% of the patients (negative T-waves in II, III, and aVF or in V3-V6), whereas troponin elevation was not present with any of the patients [median highly sensitive troponin (hs-TnT) of 9.9 ng/L, range 7.1–12.9 ng/L]. Inflammatory values like C-reactive protein (CRP) on the other hand, were markedly elevated in patients #1–3 (median CRP of 73.9 mg/dl, range 4–227 mg/dl), who presented within 2 weeks after the onset of symptoms but were normal in patients #4–5. No infectious cause for the elevated CRP could be identified in patients #1–3. COVID-19 swab testing using polymerase chain reaction was negative in all patients.

**Figure 2:**
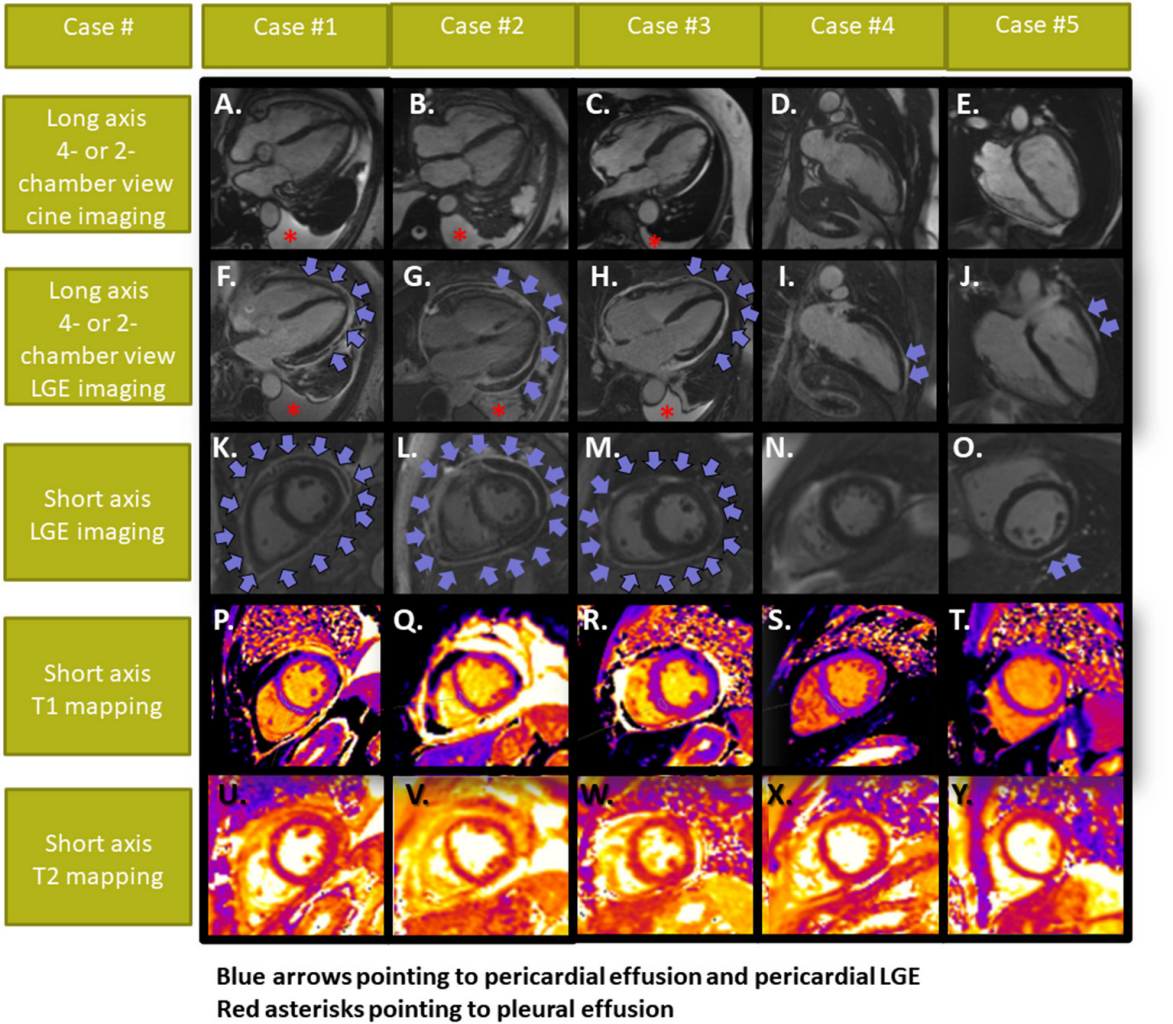
Cardiac MRI images of patients #1–5. Cine images are displayed in **(A–E)**. All patients showed pericardial LGE, which was either diffuse [**(F,K)** in patient #1; **(G,L)** in patient #2 and **(H,M)** in patient #3] or focal [**(I, N)** in patient #4 and **(J,O)** in patient #5], whereas myocardial LGE or elevated T1- and T2-values **(P–Y)** were not present with any of our patients. Pericardial and pleura effusion was present in patients #1–3. Patient #2 developed signs of a pericardial tamponade and underwent urgent pericardiocentesis (arrows depicting pericardial effusion and LGE in cases #1–3 and pericardial LGE without effusion in cases #4,5; asterisks pointing to the pleura effusions in cases #1–3).

Echocardiography and pleural sonography revealed pericardial and pleura effusion in patients #1–3, while left-ventricular (LV)-function was normal with all patients. All patients underwent CMR, which confirmed diagnosis of polyserositis with pericardial and pleura effusion in patients #1–3. LV-ejection fraction was normal in all patients (median LV-ejection fraction of 65%, range 59–69%). Using late gadolinium enhancement (LGE), pericardial enhancement was observed in all patients, being diffuse in patients #1–3 and regional in patients #4–5 (Figure 2). T1 and T2 values were within normal range in all 5 patients. In addition, epicardial, intramyocardial or endocardial LGE was not present in any of the patients. Treatment with colchicine was administrated in all patients, resulting in clinical improvement in patients #1–5. In patients #2 and #3, who presented with more severe symptoms, additional treatment with corticosteroids was necessary in both patients, whereas in patient #2 paracentesis of the pericardial effusion was necessary due to hemodynamic instability and compression of the right ventricle at admission. CMR was performed after paracentesis in this patient. The pericardial effusion was serous, rich in neutrophilic granulocytes and without tumour cells. All patients so far exhibited good mid-term outcomes without major adverse events at 3–6 months of follow-up, all remaining under close surveillance.

## Discussion

Our case series reports on five patients who presented with acute pericarditis without myocardial damage in close temporal association to the administration of SARS-CoV-2 vaccines. Although, the present data does not definitely prove causality between the vaccine and the observed acute pericarditis, it should raise the awareness throughout the clinical scientific community to carefully register such potential adverse side effects. Epidemiological studies would be necessary in this context to prove the statistical probability of such adverse effects after SARS-CoV-2 vaccination.

For the diagnosis of acute pericarditis, several clinical, ECG, echocardiography and CMR data were considered in our study. Since CMR alone may be inconclusive for the diagnosis of isolated pericarditis, current guidelines recommend the consideration of multiple diagnostic modalities and of the clinical presentation of the patients, to establish the correct diagnosis (8, 9). Despite these considerations, diagnosis of an isolated acute pericarditis may still be challenging or even remain controversial in some cases. In this regard, the subsequent clinical course and response to anti-inflammatory treatments may further help supporting the initial suspicion. Finally, it underlies the clinical judgement of the treating physician to establish the final diagnosis of this clinical entity.

The current cases series may serve as hypothesis generating for patient characteristics prone to develop acute isolated pericarditis after SARS-CoV-2 vaccination. In this respect, all affected patients had multiple doses of various vaccines and were older than those with vaccine-related myocarditis in current reports (5, 6). If multiple vaccine doses and increasing age are true risk factors for SARS-CoV-2 vaccination isolated pericarditis merits further investigation in future epidemiological studies. Interestingly, pericarditis was frequently associated by concomitant pleura effusion in our case series. Possibly the vaccination seems to have triggered a systemic inflammatory syndrome, manifesting as a secondary polyserositis, which can also be caused by severe systemic inflammation, for e.g., due to SARS-CoV-2 infection (10, 11). It should be noted, however, that pericardial abnormalities by CMR were more prominent in patients #1-3 compared to #4-5, where changes were regional and subtle. This may by attributed to the longer duration between onset of symptoms and presentation of the patients, as well as the milder form of clinical manifestation.

Even if future studies confirm the occurrence of acute isolated pericarditis or polyserositis after SARS-CoV-2 vaccination, such adverse effects can be considered as rare. However, clinicians need to be aware of such potential adverse effects since such patients benefit from prompt diagnosis and anti-inflammatory treatment. Thus, our patients could be treated successfully, and in all cases without short-term residues. Regarding these aspects, the risk of such rare and possibly reversible adverse effects should be balanced against the benefits of protecting against severe COVID-19 related complication and seem to clearly outweigh such risks in this context.

## Data availability statement

The raw data supporting the conclusions of this article will be made available by the authors, without undue reservation.

## Ethics statement

The studies involving human participants were reviewed and approved by University of Heidelberg. The patients/participants provided their written informed consent to participate in this study. Written informed consent was obtained from the individual(s) for the publication of any potentially identifiable images or data included in this article.

## Author contributions

MO and GK designed the study, performed the analysis, wrote, reviewed the manuscript, and provided important intellectual input. SG performed the acquisitions, reviewed the manuscript, and provided important intellectual input. SG, MH, MO, GK, and SH reviewed the manuscript and provided important intellectual input. All authors contributed to the article and approved the submitted version.

## Funding

For the publication fee we acknowledge financial support by Deutsche Forschungsgemeinschaft within the funding programme Open Access Publikationskosten as well as by Heidelberg University.

## Conflict of interest

The authors declare that the research was conducted in the absence of any commercial or financial relationships that could be construed as a potential conflict of interest.

## Publisher's note

All claims expressed in this article are solely those of the authors and do not necessarily represent those of their affiliated organizations, or those of the publisher, the editors and the reviewers. Any product that may be evaluated in this article, or claim that may be made by its manufacturer, is not guaranteed or endorsed by the publisher.
